# A 12-Bit 2 GS/s Single-Channel High Linearity Pipelined ADC in 40 nm CMOS

**DOI:** 10.3390/mi14071291

**Published:** 2023-06-24

**Authors:** Feitong Wu, Xuan Guo, Hanbo Jia, Xiuheng Wu, Zeyu Li, Ben He, Danyu Wu, Xinyu Liu

**Affiliations:** 1 Institute of Microelectronics of Chinese Academy of Sciences, Beijing 100029, China; 2 University of Chinese Academy of Sciences, Beijing 100049, China

**Keywords:** single channel, pipelined analog-to-digital converter(ADC), high linearity, wide bandwidth

## Abstract

This paper presents a single-channel 12-bit, 2 GS/s pipelined analog-to-digital converter (ADC) for wideband sampling receivers. The design adopts a novel source follower input buffer with multiple feedback loops to improve sample linearity and extend bandwidth. Additionally, an improved two stages charge pump amplifier topology is introduced, which doubles the Gain Bandwidth Product (GBW) without consuming additional power. To address the back-end ADC and background calibration, a multi-level dither strategy is employed, utilizing a new high-speed and low-cost uniform distribution pseudorandom code generator. The prototype ADC fabricated in 40 nm CMOS process achieves 68.24 dB SFDR up to Nyquist frequency with a sampling rate of 2 GS/s. Measurement results demonstrate a bandwidth exceeding 5 GHz, resulting in a Schreier FOMs of 152.4 dB.

## 1. Introduction

The demand for systems with faster transmission rates and higher signal quality has experienced significant growth as millimeter-wave wireless, radio, and cellular communications continue to expand. This expansion has necessitated the development of broadband ADCs with bandwidths beyond GHz and high accuracy for digitizing Intermediate Frequency (IF) signals or sampling directly at Radio Frequency (RF) [1,2,3,4,5]. In the field of communications protocols, radar scanning, and electronic measurements, the detection and decoding of weak signals play a central role in differentiating system performance. Spurious-Free Dynamic Range (SFDR) represents the minimum power signal that can be distinguished from large interfering signals, making it critical to distinguish signals from other noise and spurious frequencies. Among various conversion architectures, such as flash, folding, and successive approximation registers (SAR), pipelined conversion designs offer the most favorable balance between conversion speed, resolution, and suitability for wide bandwidths, high precision, and high sampling rates.

Interleaving enables the concurrent operation of multiple ADC cores, thereby achieving a higher sample rate than a single core [6,7]. The majority of time-interleaving (TI) ADCs introduced in recent years achieved > 2 GS/s and 12+ bit resolution [8,9,10]. The utilization of multi-channel interleaving, particularly SAR ADCs and pipelined SAR ADCs, imposes substantial system-level costs. When many subchannels are interleaved, the input bandwidth is reduced by the intricate construction of input and clock networks. Moreover, there are slight differences in phase, offset, gain, and bandwidth between the inputs of each of these cores. As a result, new interleaving artifacts and image spurs can be introduced into the frequency spectrum, thereby diminishing the wideband SFDR of the ADC, which is not suitable for broadband applications.

There are several challenges in the design of high-speed ADCs. First, the front-end section plays a crucial role in determining the overall performance. For example, in a multi-channel time-interleaved SAR ADC is limited by the high input capacitance, and if its input front-end section is not properly layered and interleaved, the overall performance will be severely degraded, regardless of the back-end ADC section’s performance [10,11]. Alongside the matching network and interface part, the input buffer is one of the most important parts. Secondly, despite the remarkable progress in CMOS technology, which has led to a reduction in parasitic capacitance of MOSFETs and increased the cutoff frequency, the performance and speed of pipelined ADCs still face limitations imposed by amplifiers, especially when operating at GS/s sampling rate. The inherent gain of transistors is decreasing, impacting the design of high-gain operational amplifiers and reducing the linearity of ADCs. Although modern technologies can reduce amplifier signal stabilization time and increase conversion rates, the diminishing inherent gain of the device with decreasing feature size, due to process development, making it much more difficult for the amplifier to achieve gain accuracy. It has become a design challenge to increase the gain and bandwidth of operational amplifiers without significantly increasing the power consumption. Recent research has proposed various approaches based on ring amplifiers [12,13]. However, the research point focuses on reducing power consumption, and the application is predominantly in the low speed range. Despite the ring amplifier’s excellent performance, it currently exhibits vulnerability to process fluctuations and weak robustness, particularly when the supply voltage fluctuates, rendering it inadequate to meet industrial standards.

Dither injection can be used to reduce harmonics and noise generated by jitter signals within the ADC, thereby optimizing the spectrum to improve the ADC’s output performance. There are two different bandwidth-based jitter methods. One is wideband dithering, which utilizes a dither DAC and a pseudorandom number (PN) generator to generate the dithered signal. The jitter signal added to the input must be digitally removed from the output to prevent degradation of the signal-to-noise ratio. Another method is narrowband dithering, which involves a broadband noise source and an analog low-pass filter to generate the dither signal outside the target band. Out-of-band jitter also needs to be removed during the post-processing of the digital filter applied to the ADC output. The extensively discussed dithering techniques are based on the first method. The dithering mentioned in [14], although not limited to the bandwidth, sacrifices some available signal bandwidth and dynamic range in the frequency domain. Ali et al. [15] mentioned an injection method applicable to pipelined ADCs, enabling a dithering injection technique for residual nonlinear signals. However, the details of the specific circuit implementation are not described in the paper.

By implementing a linearity enhancement technique in this design, a 12-bit 2 Gs/s ADC was designed, fabricated, and measured. The proposed feedback loop-based input buffer ensures the speed and linearity of the entire ADC system. A two-stage amplifier with a charge pump is used to achieve high gain and high bandwidth simultaneously, and a dither signal injection logic is designed to further improve the linearity and optimize the output spectrum.

This paper is structured as follows: Section 2 discusses the circuit ideas proposed in this paper and the techniques employed to enhance linearity and speed, thereby extending the effective input bandwidth. In Section 3, we describe the implementation of these ideas in a prototype pipeline ADC and present the measurement results. Finally, Section 4 concludes this paper.

## 2. Proposed Pipelined ADC Circuit Implementation

### 2.1. Overall Circuit Structure

**Figure 1:**
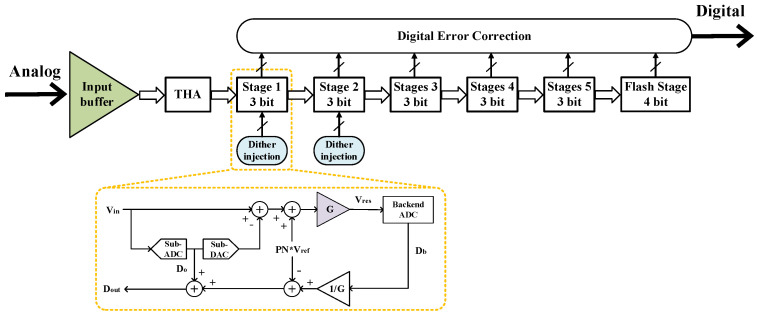
Block diagram of the proposed pipelined ADC.

Figure 1 depicts the proposed 12-bit 2 GS/s pipelined ADC architecture. When employing a sample-and-hold-less (SHA-less) amplifier structure, the SFDR performance is constrained due to aperture errors and complex input matching networks, particularly in the case of high-frequency input signals [16]. Hence, despite the additional power consumption introduced by the track-and-hold amplifier (THA), it remains a crucial component for achieving a high sampling rate in the ADC. The first two stages consist of a 3.5-bit stage with dither injection of the multiplying digital-to-analog converter (MDAC), followed by three 3-bit stages and a 4-bit flash ADC. To prevent over-ranging of the MDAC’s output voltage, redundancy is incorporated into each of the stages from 1 to 5. The first stage employs a sampling capacitance of 400 fF, the second stage uses 100 fF, and the third through fifth stages employ 50 fF sampling capacitances.

### 2.2. Input Buffer

**Figure 2:**
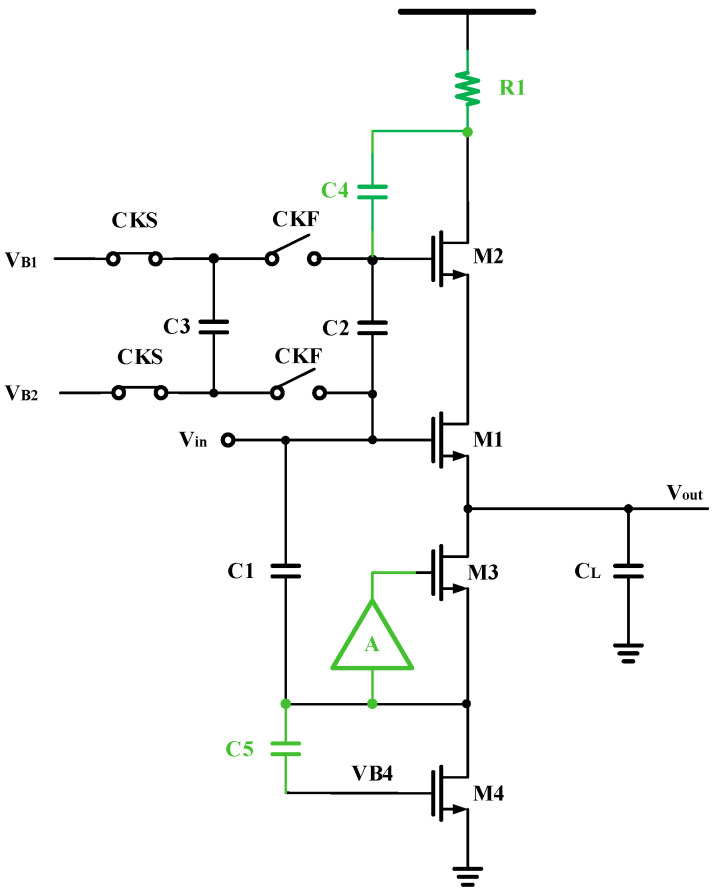
Proposed Input buffer structure.

The input buffer is often integrated before the ADC to isolate the kickback noise and package inductance. The structure adopted for the input buffer is based on the work presented in [2], as illustrated in Figure 2. The supply voltage is raised to 2.5 V to ensure a large output swing. Additionally, R1 is added to reduce the voltage burden on M1, and M2 within the output common mode voltage is set at 0.9 V. For M1:

(1) $$I_{1}=\frac{1}{2}\mu C_{ox}\frac{W}{L}{\left(V_{gs}-V_{th}\right)}^{2}\left(1+\lambda V_{ds}\right)$$

When *V_ds_* changes, *V_gs_* will also change, assuming I remains constant. To address this issue, the research [3] advises employing a switching capacitor as a level converter to maintain constant *V_ds_*. Additionally, to enhance the stability of Equation (1), it is also required to impose a limit on I while maintaining *V_ds_* at a constant value. C4 and C5 are also added to reduce current variation, creating a negative feedback loop through the capacitor connected to M4, which can partially suppress the current variation. For this structure, its voltage transfer function can be expressed as:

(2) $$\frac{v_{out}}{v_{in}}=\frac{1}{1+\frac{1}{(g_{m}+\frac{1}{Z_{out}})Z_{L}}}$$

If $g_{m}\gg \frac{1}{Z_{out}}$, then:

(3) $$\frac{v_{out}}{v_{in}}=\frac{1}{1+\frac{1}{g_{m}Z_{L}}}$$

This means that the buffer can enhance voltage following the effect by increasing both the transconductance gm and the output impedance *Z_L_*. However, raising the gm of the source stage follower is extremely challenging. Therefore, raising the output resistance is an alternative approach to enhance linearity. To increase the output resistance without affecting the output node’s parasitic capacitance, the gain boosting structure is introduced to the gate of M3 in this design, c significantly improving the linearity of the low frequency signal. The bias voltage VB4 of M4 can be artificially adjusted within a certain range. If the input Vin is a high frequency signal, VB4 can be increased moderately to alleviate the effect of reduced bandwidth.

### 2.3. Amplifier

The amplifier must have a high gain to ensure the accuracy needed in a high-resolution pipeline ADC. Gain error for pipeline ADCs with N-bit resolution should be smaller than $\frac{1}{2^{N}}$ so that:

(4) $$\Delta \approx \frac{1}{A\beta 2^{n}}<\frac{1}{2^{N}}$$

where *A* is the gain of the error amplifier, *β* is the feedback coefficient, and *n* is the quantization accuracy of this MDAC stage. For 12-bit, the first stage MDAC is set to 3-bit and *β* = 4, then the required gain should be more than 70 dB.

Similarly, the *GBW* of the amplifier also needs to satisfy:

(5) $$GBW>\frac{\left(N-n\right)\log2}{\beta \pi }\cdot Fs$$

where *Fs* is the ADC’s sampling rate, and in the case of 2G, ensure that the total *GBW* is at least 10 GHz. It is preferable to leave a substantial margin above the required values to account for manufacturing bias, which also helps to minimize gain and offset errors, as well as reduce the burden of calibration.

To attain a high gain, telescoping cascode amplifiers with a gain boost structure has been employed extensively in the past [4]. This design employs a two-stage cascaded amplifier topology, wherein the first stage utilizes a standard gain bootstrap while the second stage offers a large bandwidth. To further enhance the performance of the amplifier, an additional charge pump is introduced between the input and the conventional amplifier-based cascade device. This charge pump is driven by the non-crossover clocks CKS and CKF. By periodically charging and discharging the load tube gate capacitor to generate an additional AC signal input and the associated bias voltage. During CKF operation, the voltage difference between VB and VC is maintained across CB. CI’s value determines the ripple’s magnitude and stabilization time, necessitating CI to be significantly smaller than CBI. When the transconductance of M3 and M9 is comparable, the associated Gm actually doubles, and the nmos and pmos cascade structures become each other’s output impedance. This configuration results in an increase in the high-frequency and small-signal gain of this stage. The fixed deviation between the P- and N-terminal input devices can be maintained by multi-cycle capacitor charging and discharging, which expends the signal swing.

In the 40 nm process, achieving device mobility matching between PMOS and NMOS becomes challenging. However, by carefully selecting an appropriate bias point, the adverse impact of multipole pairing can be significantly mitigated. This means that careful design of the transistor size is required. This improvement in the cascade configuration greatly enhances the stabilization time of the amplifier.

**Figure 3:**
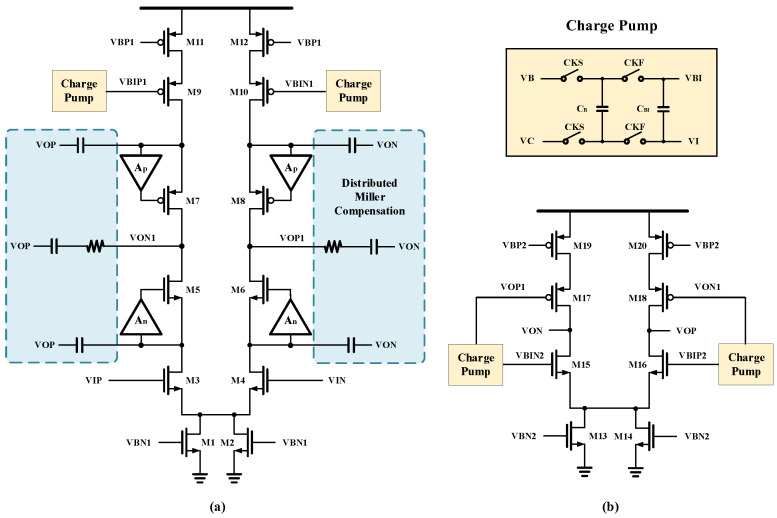
Amplifier design consists of (**a**) the first stage and (**b**) the second stage.

**Figure 4:**
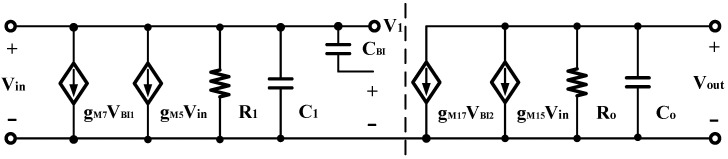
Small signal for the half circuits of the proposed structure.

To simplify layout drawing and minimize differential offset, the proposed error amplifier in Figure 3 adopts a fully symmetric design. The corresponding one-sided circuit small-signal model is displayed in Figure 4.

(6) $$A_{v1}=\frac{V_{1}}{V_{in}}\left(g_{m3}+g_{m9}\right)\left(R_{1}∥\frac{1}{sC_{1}}\right)$$

(7) $$A_{v2}=\frac{V_{2}}{V_{1}}\left(g_{m16}+g_{m18}\right)\left(R_{2}∥\frac{1}{sC_{2}}\right)$$

where *R*_1_ and *R*_2_ are the output resistances of the first and second stage output nodes:

(8) $$R_{1}=A_{n}\cdot g_{m3}r_{o1}r_{o3}∥A_{p}\cdot g_{m5}r_{o5}r_{o7}$$

(9) $$R_{2}=r_{o16}∥g_{m18}r_{o18}r_{o20}$$

The power supply voltage is 2 V, while the output common mode voltage is set to 1 V. By employing M9 and M10, the voltage pressure of M5-M8 are effectively reduced, enabling the utilization of faster device types. To uphold the optimal operating point, the amplifier actively monitors changes in process, supply, and temperature (PVT) with respect to its output and input common-mode voltages. This enables the amplifier to achieve high DC gain and a swift swing rate, effectively minimizing nonlinear error. In addition, distributed Miller compensation between the first and second stages ensures stable loop operation, providing a fast and stable response to large output variations. The THA and MDAC1 consume the largest current, while the full-stage MDAC uses the same amplifier structure that uses a scaled-down current for the final stage.

**Figure 5:**
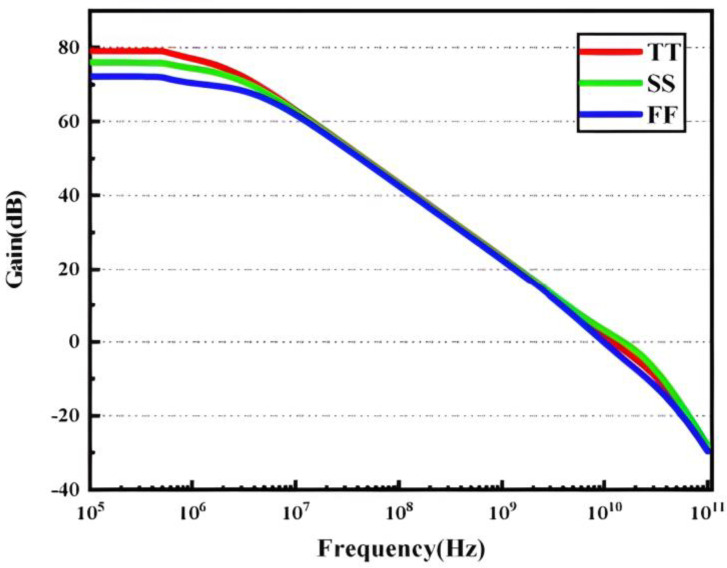
Loop gain response of the two-stage amplifier.

**Figure 6:**
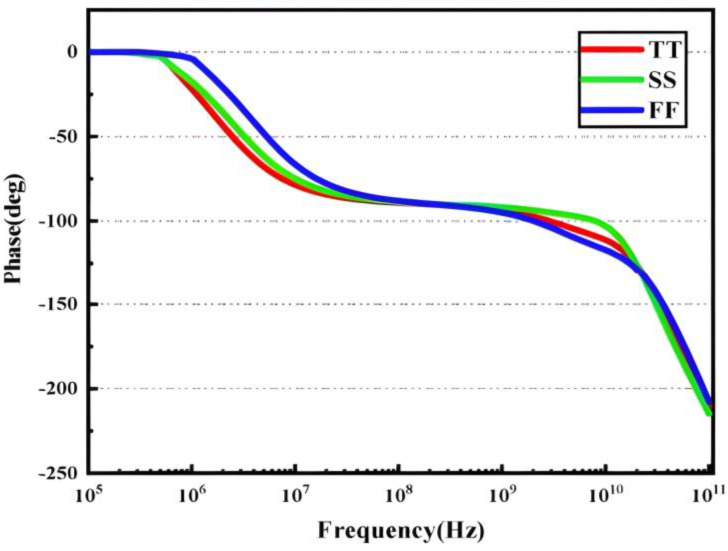
Phase of the two-stage amplifier.

The developed amplifier’s loop frequency characteristics are simulated under various PVT settings to ensure its stability, and the results are displayed in Figure 5 and Figure 6. The phase margin exceeds 60, the GBW reaches 11.002 GHz, and the low-frequency loop gain is 72.07 dB.

### 2.4. Comparator

**Figure 7:**
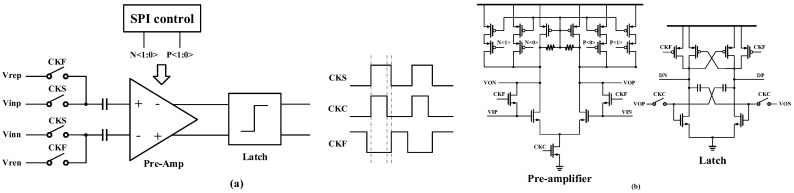
Comparator design (**a**) structure and time diagram, (**b**) preamplifier and latch.

For comparator design, the offset is an inconvenient factor in addition to speed [17]. Due to the limitations of the chip fabrication process, the presence of offset in the comparator becomes inevitable. Consequently, effective offset reduction emerges as a key focal point in the design process. One way of eliminating the offset is to short the input to the comparator to achieve a full reset by setting an additional cycle timing control. Limited by the sampling rate, the comparator’s normal operating state may be affected if the timing is not set properly. Comparators tend to adopt a circuit structure that combines a preamplifier and a latch to achieve high speed, as shown in Figure 7a. Two transistors act as switches driven by the clock signal CKF in Figure 7b and are utilized to connect the input and output of the pre-amplifier [18]. During the CKS clock signal’s invalid state, a threshold voltage, associated with the preamplifier offset, is stored across the front-end sampling capacitor, enabling the self-calibration function and ensuring the next cycle.

The preamplifier incorporates a resistor that serves a dual purpose. Firstly, it stabilizes the common-mode voltage between the two outputs. Secondly, it increases the output impedance, and subsequently, the gain. When the clock signal CKC becomes active, the preamplifier sends the amplified signal to the latch assembly. The latch uses a direct cross-coupled positive feedback structure to limit the output swing.

**Figure 8:**
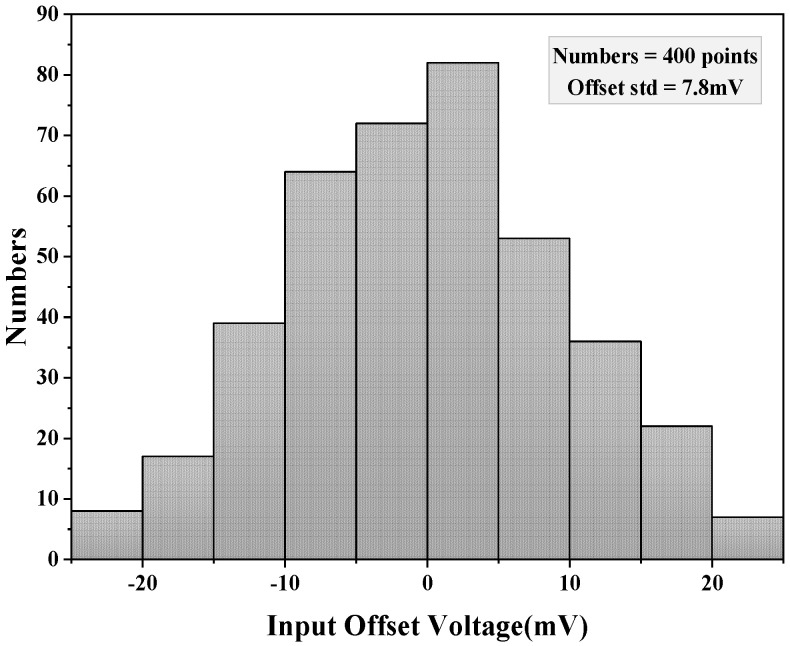
Monte Carlo simulation results.

An engineered trim method is used on the comparator to enable manual adjustment for compensating offsets resulting from manufacturing during the final test. Control ports such as P<1:0> and N<1:0> are added to the gate of the preamplifier output load for external offset calibration. Therefore, each comparator incorporated within the developed ADC possesses the capability to modify its offset voltage using SPI command control. The adjustment level can be determined by observing the residual error curve of the MDAC at different levels. It is possible to control the working state of the circuit, reducing the offset voltage without affecting the performance of the circuit. The results of the Monte Carlo simulation are depicted in Figure 8 and the comparator’s offset voltage is 7.8 mV.

### 2.5. Dither Injection

**Figure 9:**
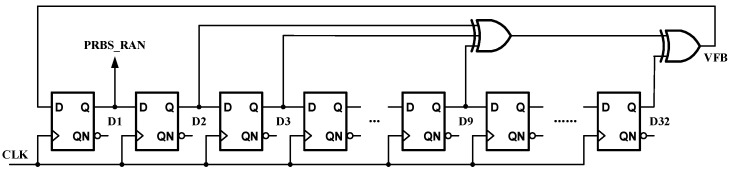
A random code implementation.

A pseudo-random sequence and switches are used for dither injection. Therefore, within the first two MDAC stages, the pseudo-random binary sequence (PRBS) generator is used. This generator utilizes a linear feedback mechanism, employing a cascade of multiple flip-flops, to produce a periodic distribution of zeros and ones, as well as an autocorrelation characteristic similar to white noise in the frequency spectrum. As an example, a 32-bit PRBS generating circuit implementation is shown in Figure 9.

**Figure 10:**
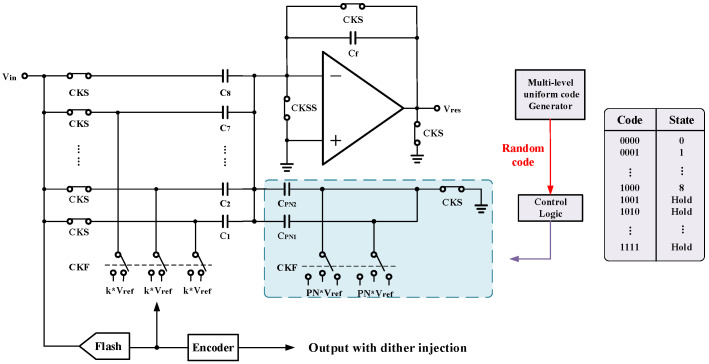
The structure of MDAC with dither injection.

The single-ended circuit structure of MDAC1 is shown in Figure 10. The sample capacitor of the MDAC1 is C1–C8 with a value of Cu, and the feedback capacitor is Cf with a value of 2 Cu. The sub-DAC uses the CPN capacitor to implement the pseudo-random noise injection. The switches connected the capacitors to reference voltage are controlled by PN code. In MDAC1, CPN1 is set to three times CPN2.

The reference voltage state connected to the injected capacitors CPN1 and CPN2 is directly influenced by each PN code (−1, 0, or 1). Each of the two capacitors has three possible connection states, resulting in a total of nine states, which means that the number of levels of the multi-level dither is nine. Accordingly, only one capacitor is set in MDAC2, so there are three states. An intuitive result of dithering injection is the thickening of the residual curve flip line, accompanied by random fluctuations in the raw output-weighted waveform. These fluctuations introduce randomness that disrupts the periodic effect in the original signal, dispersing high-frequency harmonics to the noise floor and reducing their peaks. As a result, the linearity is improved.

**Figure 11:**
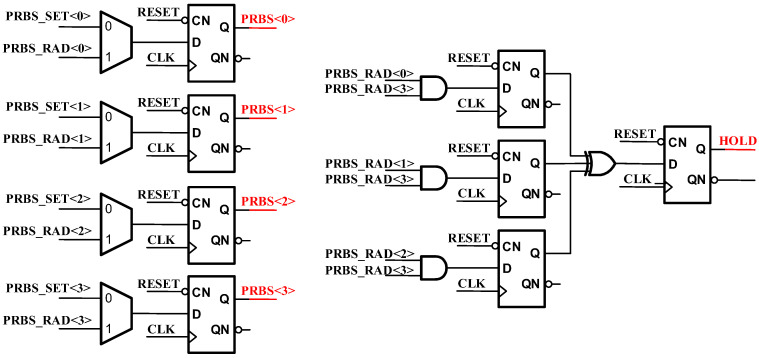
Random code logic.

Additionally, two modes of operation exist for the PRBS code: manual setting mode and automatic setting mode, as shown in Figure 11. The automatic reset command corresponds to PRBS_RAN<3:0>, while the manual reset command corresponds to PRBS_SET<3:0>. The manual setting was added to facilitate the verification of the actual level of jitter injection in the test. When PRBS_RAN<3:0> is valid, different PRBS code combinations indicate different PN code states, and state 0–state 8 correspond to the nine connection states above. Furthermore, HOLD states are to maintain the connection method from the previous cycle, ensuring an equal chance for each state to occur.

## 3. Experimental Results and Discussion

**Figure 12:**
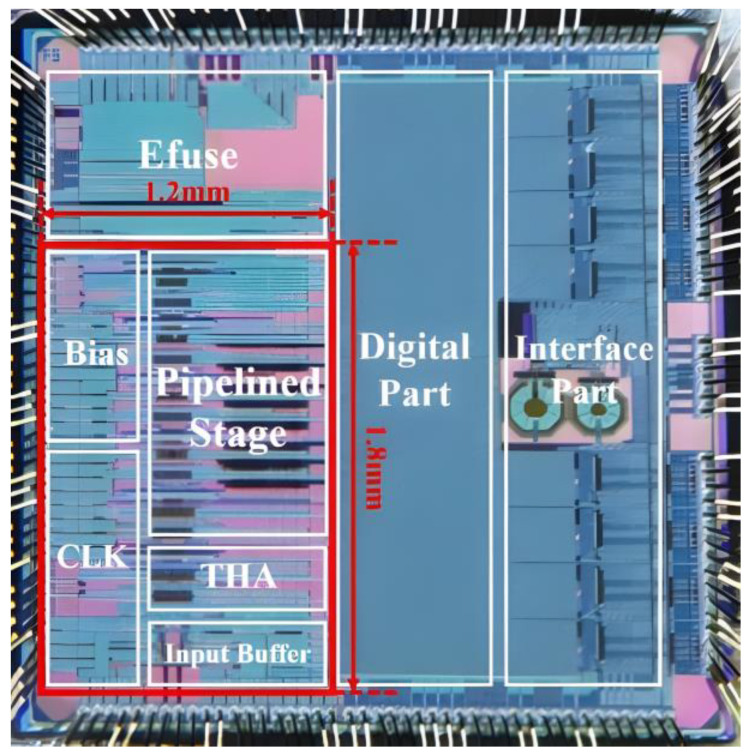
Die photo.

The presented pipelined ADC has been designed and fabricated with 40 nm CMOS technology. The die micrograph with a size of 3 mm × 3 mm is shown in Figure 12. The analog part of the ADC core occupies approximately 25% of the 1.2 mm × 1.8 mm area. 

**Figure 13:**
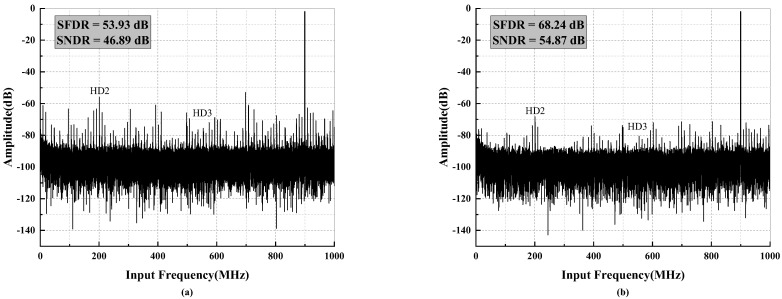
Measured output spectrum when fin = 900 MHz with (**a**) calibrations OFF (**b**) calibrations ON.

**Figure 14:**
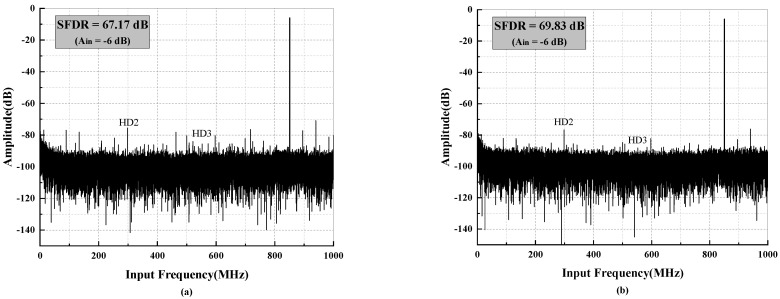
Measured output spectrum when A_in_ = −6 dB with (**a**) dither switched off (**b**) dither switched on.

Figure 13 presents the fast Fourier transform (FFT) spectrum obtained from measuring the ADC sampling a 2 GHz input signal at 900 MHz. The spectrum illustrates the performance limits of HD2 and HD3. After calibration, as depicted in Figure 13b, the spurious signals are mitigated, resulting in an improved spurious-free dynamic range (SFDR) of 68.24 dB, while the achieved signal-to-noise and distortion ratio (SNDR) reaches 54.87 dB. As the input signal decreases, HD2 and HD3 generally improve as the square and tube of the signal reduction. Consequently, the SFDR can quickly become limited by residual spurs. When the dithering mode is enabled, as shown in Figure 14, the SFDR further increases from 67.17 dB to 69.83 dB, while SNDR experiences a slight decrease.

**Figure 15:**
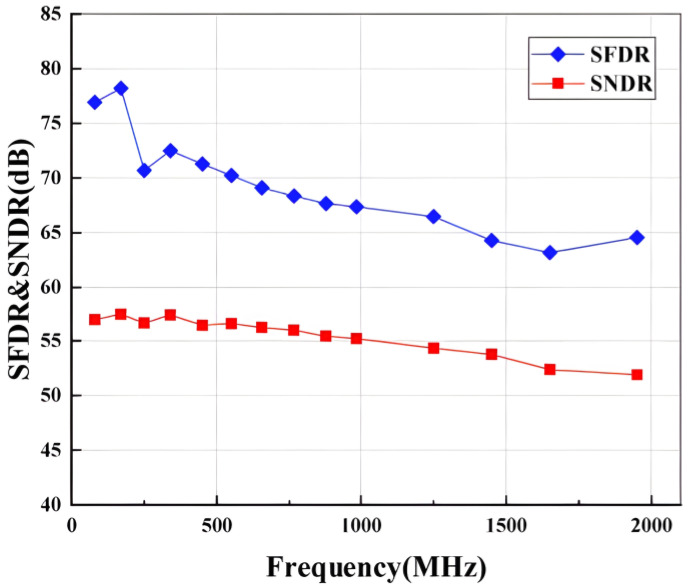
Performance comparison at 2 GS/s sampling rate, versus input signal frequency.

**Figure 16:**
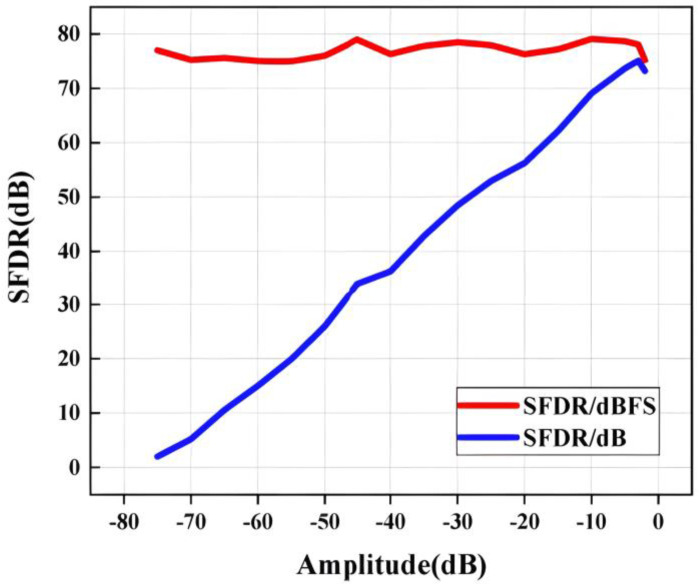
Measured performance versus input amplitude.

Figure 15 presents the measured the SNDR and SFDR of the ADC versus input frequency. This ADC achieves an SFDR/SNDR of 78 dB and 58 dB at low input frequency and remains approximately 68 and 55 dB at Nyquist frequency, respectively. Figure 16 shows the measured SFDR versus input signal amplitude, where the input signal amplitude varies from −1 dB to −75 dB. The SFDR is held stable above 75 dBFS with good linearity as the input signal amplitude varies.

**Figure 17:**
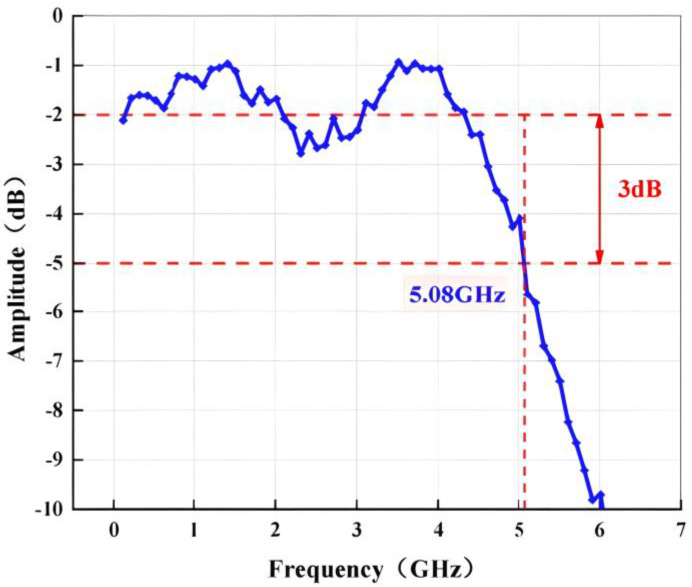
The bandwidth of the proposed pipelined ADC.

Figure 17 shows the bandwidth of the ADC, including the degradation due to input bonding wires. To avoid exceeding the full amplitude range, we set −2 dB as the starting reference. The measurement results show the BW exceeds 5 GHz, which can adequately meet the needs of high-speed broadband applications. The ADC core consumes 895 mW of power. Specifically, the wideband input buffer accounts for 156 mW, the THA section consumes 125 mW, and the first stage MDAC utilizes 138 mW. The remaining power consumption is attributed to the back-end MDAC stages, flash stage and clock generator.

Table 1 describes the proposed ADC’s performance and contrasts it with prior state-of-the-art high-resolution ADCs that operate at speeds of up to 1 GS/s. The proposed ADC clearly benefits from linearity without making significant energy consumption ratio sacrifices. Our design has an optimal SFDR due to the many proposed linearity improvement techniques. Moreover, based on the achieved sufficient bandwidth, the presented ADC gives a schreier FoM of 152.4 dB, which outperforms the majority of pipelined ADCs in Table 1. Overall, this design demonstrates a 12-bit 2 GS/s ADC with state-of-the-art performance implemented in a 40 nm process.

## 4. Conclusions

A 2 GS/s, 12-bit, single-channel pipelined ADC in 40 nm technology has been introduced. In order to provide higher linearity, a new input buffer with multiple feedback loops has been proposed. It further increases the bandwidth. The use of a second operational amplifier with the charge pump structure further enhances the gain to meet high resolution requirements without increasing power consumption. Moreover, by using the dither injection technique, the SFDR can be further improved and the spectrum can be optimized. The single channel pipelined ADC has an SFDR of up to 78 dB at low frequency signal inputs, and 68.24 dB at the Nyquist frequency. It also achieves a FoMs of 152.4 dB at the Nyquist frequency. The measurement result demonstrates the effectiveness of the proposed techniques in improving linearity and effective bandwidth in advanced high-speed ADC technology.

## Figures and Tables

**Table 1:** Comparison of Other Design.

Specification	[4]	[6]	[7]	[10]	[19]	[20]	This Work
Structure	Pipelined	Pipelined	Pipelined	Pipelined	Pipelined-SAR	Time domain	Pipelined
Interleaving	Yes	Yes	Yes	Yes	Yes	No	No
Technology (nm)	40	65	28	16	65	28	40
Resolution (bit)	12	-	12	13	11	10	12
Sampling rate (GHz)	3	4	1.6	5	1	2.5	2
SNDR@Nyq. (dB)	58	55.5	57	57	55.9	50.1	54.87
SFDR@Nyq. (dB)	-	64	66	61.9	60.5	58.5	68.24
Power (mW)	500 *	2200 *	1950	641	230 *	31.5 *	895 *
FoMs@Nyq. (dB)	145.8	145.1	148.4	152.9	149.4	156	152.4
Area (mm^2^)	0.4	12	3.8	1.1	2.5	0.074	2.16

FoMs = SNDR + 10 log(BW/Power); * ADC core consumption.

## Data Availability

Not applicable.

## References

[1] Wang L., Marc-Andre L., Anthony C.C. (2017). A 4-GS/s single channel reconfigurable folding flash ADC for wireline applications in 16-nm FinFET. IEEE Trans. Circuits Syst. II Express Briefs.

[2] Ali A.M., Dinc H., Bhoraskar P., Dillon C., Puckett S., Gray B., McShea M. (2014). A 14 bit 1 GS/s RF sampling pipelined ADC with background calibration. IEEE J. Solid-State Circuits.

[3] Ali A.M., Dinc H., Bhoraskar P., Puckett S., Morgan A., Zhu N., Taylor G. A 14-bit 2.5 GS/s and 5 GS/s RF sampling ADC with background calibration and dither. Proceedings of the 2016 IEEE Symposium on VLSI Circuits (VLSI-Circuits).

[4] Chen C.Y., Wu J., Hung J.J., Li T., Liu W., Shih W.T. (2012). A 12-Bit 3 GS/s Pipeline ADC With 0.4 mm 2 and 500 mW in 40 nm Digital CMOS. IEEE J. Solid-State Circuits.

[5] Devarajan S., Singer L., Kelly D., Pan T., Silva J., Brunsilius J., Manganaro G. (2017). A 12-b 10-GS/s interleaved pipeline ADC in 28-nm CMOS technology. IEEE J. Solid-State Circuits.

[6] Straayer M., Bales J., Birdsall D., Daly D., Elliott P., Foley B., Wang X. 27.5 A 4 GS/s time-interleaved RF ADC in 65 nm CMOS with 4 GHz input bandwidth. Proceedings of the 2016 IEEE International Solid-State Circuits Conference (ISSCC).

[7] El-Chammas M., Li X., Kimura S., Maclean K., Hu J., Weaver M., Gindlesperger M., Kaylor S., Payne R., Sestok C.K. (2014). A 12 bit 1.6 GS/s BiCMOS 2× 2 hierarchical time-interleaved pipeline ADC. IEEE J. Solid-State Circuits.

[8] Lin C.Y., Yen-Hsin W., Tai-Cheng L. (2018). A 10-bit 2.6-GS/s time-interleaved SAR ADC with a digital-mixing timing-skew calibration technique. IEEE J. Solid-State Circuits.

[9] Wang X., Wang C., Li F., Wang Z. A Low-Power 12-bit 2 GS/s Time-Interleaved Pipelined-SAR ADC in 28 nm CMOS Process. Proceedings of the 2018 IEEE International Symposium on Circuits and Systems (ISCAS).

[10] Vaz B., Verbruggen B., Erdmann C., Collins D., Mcgrath J., Boumaalif A., Cullen E., Walsh D., Morgado A., Mesadri C. A 13 Bit 5 GS/S ADC with time-interleaved chopping calibration in 16 NM FinFET. Proceedings of the 2018 IEEE Symposium on VLSI Circuits.

[11] Wang G., Sun K., Zhang Q., Elahmadi S., Gui P. (2019). A 43.6-dB SNDR 1-GS/s 3.2-mW SAR ADC with background-calibrated fine and coarse comparators in 28-nm CMOS. IEEE Trans. Very Large Scale Integr. VLSI Syst..

[12] Lim Y., Michael P.F. (2015). A 100 MS/s, 10.5 bit, 2.46 mW comparator-less pipeline ADC using self-biased ring amplifiers. IEEE J. Solid-State Circuits.

[13] Lagos J., Hershberg B.P., Martens E., Wambacq P., Craninckx J. (2019). A 1-GS/s, 12-b, single-channel pipelined ADC with dead-zone-degenerated ring amplifiers. IEEE J. Solid-State Circuits.

[14] Gonzalez-Diaz V.R., Garcia-Andrade M.A., Flores-Verdad G.E., Maloberti F. (2010). Efficient dithering in MASH sigma-delta modulators for fractional frequency synthesizers. IEEE Trans. Circuits Syst. I Regul. Pap..

[15] Ali A.M., Morgan A., Dillon C., Patterson G., Puckett S., Bhoraskar P., Sneed R. (2010). A 16-bit 250-MS/s IF sampling pipelined ADC with background calibration. IEEE J. Solid-State Circuits.

[16] Dong-Young C. (2004). Design techniques for a pipelined ADC without using a front-end sample-and-hold amplifier. IEEE Trans. Circuits Syst. I Regul. Pap..

[17] Razavi B., Bruce A.W. (1992). Design techniques for high-speed, high-resolution comparators. IEEE J. Solid-State Circuits.

[18] Zheng X., Wang Z., Li F., Zhao F., Yue S., Zhang C., Wang Z. (2016). A 14-bit 250 MS/s IF sampling pipelined ADC in 180 nm CMOS process. IEEE Trans. Circuits Syst. I Regul. Pap..

[19] Liu Q., Wei S., Joseph S.C. (2018). A 1-GS/s 11-bit SAR-assisted pipeline ADC with 59-dB SNDR in 65-nm CMOS. IEEE Trans. Circuits Syst. II Express Briefs..

[20] Liu M., Zhang C., Liu S., Li D. (2021). A 10-bit 2.5-GS/s two-step ADC with selective time-domain quantization in 28-nm CMOS. IEEE Trans. Circuits Syst. I Regul. Pap..

